# Zfp207 is a Bub3 binding protein regulating meiotic chromosome alignment in mouse oocytes

**DOI:** 10.18632/oncotarget.9310

**Published:** 2016-05-11

**Authors:** XiaoXin Dai, Hao Xiong, Mianqun Zhang, Shaochen Sun, Bo Xiong

**Affiliations:** ^1^ College of Animal Science and Technology, Nanjing Agricultural University, Nanjing, China; ^2^ The First Clinical Medical College, School of Medicine, Nanchang University, Nanchang, China

**Keywords:** oocyte meiosis, Zfp207, chromosome alignment, K-MT attachment, aneuploid eggs, Pathology Section

## Abstract

Zinc finger proteins are a massive, diverse family of proteins that serve a wide variety of biological functions. However, the roles of them during meiosis are not yet clearly defined. Here, we report that Zfp207 localizes at the kinetochores during mouse oocyte meiotic maturation. Depletion of Zfp207 leads to a significantly higher proportion of impaired spindle organization and misaligned chromosomes in oocytes. This is coupled with the defective kinetochore-microtubule attachments, and resultantly increasing incidence of aneuploid metaphase II eggs. The precocious polar body extrusion and escape of metaphase I arrest induced by nocodazole treatment in Zfp207-depleted oocytes indicates that Zfp207 is essential for activation of SAC (Spindle Assembly Checkpoint) activity. Notably, we find that Zfp207 binds to Bub3 to form a complex and maintains its protein level in oocytes, and that overexpression of Bub3 is able to partially rescue the occurrence of aneuploid eggs in Zfp207-depleted oocytes. Collectively, we identify Zfp207 as a novel Bub3 binding protein in oocytes which plays an important role in controlling meiotic chromosome alignment and SAC function.

## INTRODUCTION

High-fidelity chromosome segregation ensures proper distribution of genetic material during cell division in both mitosis and meiosis [1]. Segregation errors during mitosis in somatic cells contribute to the development and progression of cancer, and segregation errors during meiosis in germ cells lead directly to miscarriages, birth defects and genetic disorders [2, 3]. To achieve faithful chromosome segregation, eukaryotic cells develop a high-fidelity surveillance system referred to as SAC (spindle assembly checkpoint) to prevent chromosome missegregation and aneuploidy by delaying anaphase onset until all kinetochores are successfully attached to the spindle microtubules with the proper tension at the metaphase plate [4–8].

SAC is mainly composed of the members of Bub and Mad families. Among them Mad2, BubR1 and Bub3 comprise the soluble Mitotic Checkpoint Complex (MCC) which inhibits the activation of anaphase-promoting complex/cyclosome (APC/C) by targeting APC/C's cofactor Cdc20, and delays the metaphase-anaphase transition until correct kinetochore-microtubule attachment is established [3, 9, 10]. Once chromosome is properly aligned at metaphase plate with appropriate tension by the spindle, SAC pathway is shut down and Cdc20 is released to activate APC/C, which then ubiquitinates Securin and Cyclin B, leading to the activation of Separase to remove the Cohesin complex from chromosome and onset of anaphase [9, 11, 12].

Chromosome segregation during cell division is facilitated by the kinetochores. When microtubules are not bound, they prevent cell cycle progression by generating the SAC signal. The kinetochore, assembled from more than 90 proteins at the centromere, is a large multiprotein structure that is often divided up into three layers: inner, central, and outer [13, 14]. The inner proteins are associated with the DNA and are linked to the outer layer by the central layer, and the outer kinetochore proteins are responsible for capturing microtubules and recruiting SAC components [7, 15–19]. Bub1, BubR1 and Bub3 are three core SAC proteins that are required for correct chromosome alignment and kinetochore-microtubule attachment. They form two heterodimers ‘Bub1-Bub3’; and ‘BubR1-Bub3’; at the kinetochores to exert the function [8, 20]. Bub3 recruits Bub1 and BubR1 by directly binding to a highly conserved GLEBS domain in Bub1 or BubR1 [8, 21, 22].

BuGZ (Bub3-interacting GLEBS-motif-containing ZNF207) /Znf207/Zfp207 is a zinc finger protein that also contains a GLEBS motif [16]. Recent studies have shown that BuGZ uses its GLEBS domain directly binds to and stabilizes Bub3 during interphase and mitosis [11, 14, 16]. As a Bub3-binding partner and chaperone, BuGZ promotes kinetochore-microtubule interaction, chromosome alignment, and mitotic progression in cancer cells [11] [14, 16]. Although it has been identified as a novel regulator of chromosome alignment in mitotic cells, its accurate role in meiosis has not yet been defined.

In the present study we provide a body of evidence demonstrating that Zfp207 is required for normal spindle organization, correct chromosome alignment, proper kinetochore-microtubule attachment, and maintenance of euploidy during mouse oocyte meiotic maturation. We also find that Zfp207 is implicated in promoting these events through, at least partially, regulation of Bub3.

## RESULTS

### Zfp207 localizes at the kinetochores in mouse oocytes

**Figure 1:**
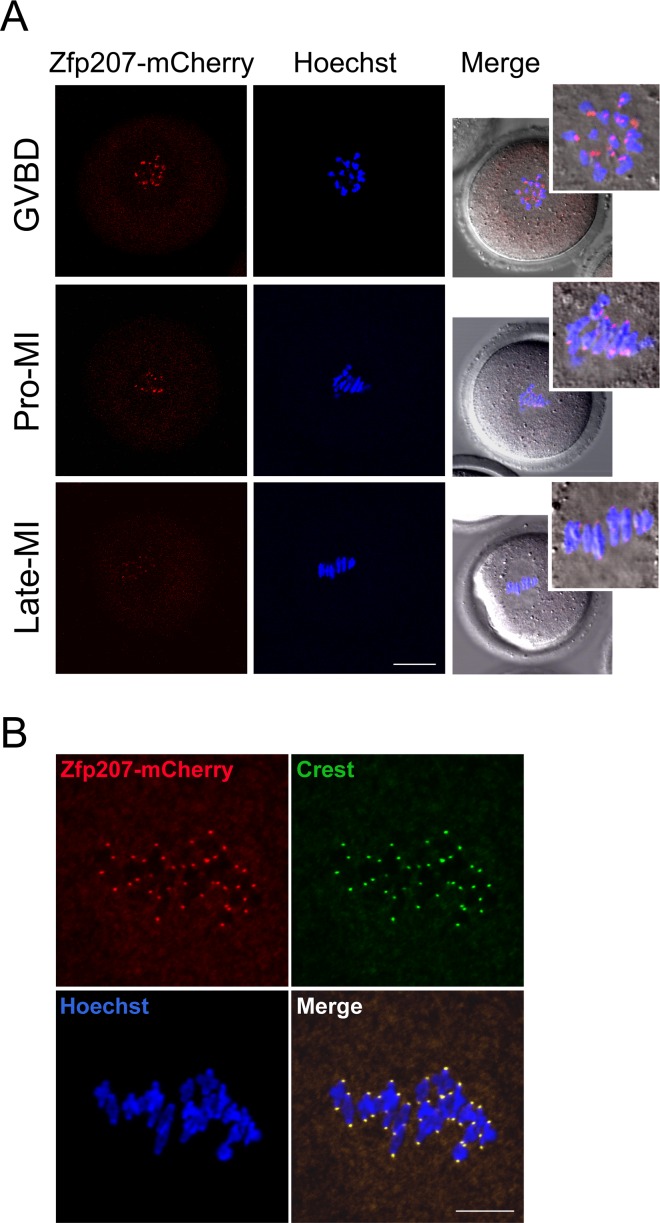
Localization of Zfp207 during mouse meiotic maturation **A.** cRNA of Zfp207-mCherry was microinjected into GV oocytes which were then cultured to various developmental stages. mCherry signals were acquired under the confocal microscope at 594 nm laser. Chromosomes were counterstained with Hoechst. GVBD, oocytes at germinal vesicle breakdown stage; Pro-MI, oocytes at first prometaphase stage; Late-MI, oocytes at late stage of first metaphase. Scale bar, 20μm. **B.** Zfp207-mCherry expressing oocytes were immunostained with kinetochore marker Crest and then counterstained with Hoechst. Scale bar, 5μm.

To examine the localization of Zfp207 during meiotic maturation in mouse oocytes, we tried several commercially available antibodies to perform the immunofluorescent analysis, but all of them were not working in oocytes. Therefore we made a construct to fuse the fluorescent tag mCherry to the C-terminus of Zfp207 and *in vitro* transcribed it into cRNA. The result of cRNA microinjection showed that Zfp207-mCherry was present at the end of chromosomes from GVBD till late metaphase I stage (Figure 1A). This localization pattern is quite similar to that of kinetochore proteins, thus we immunostained Zfp207-mCherry expressing oocytes with kinetochore marker Crest, and they indeed exhibited the overlapping fluorescent signals in the oocytes (Figure 1B), indicating that Zfp207 is localized at the kinetochores during meiosis.

### Zfp207 modulates meiotic spindle assembly and chromosome alignment in oocytes

**Figure 2:**
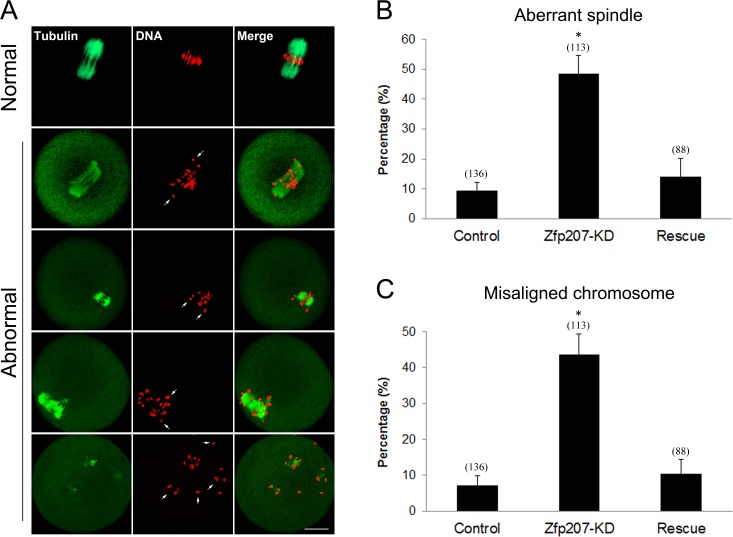
Depletion of Zfp207 impairs spindle formation and chromosome alignment in mouse oocytes **A.** Representative images of normal and abnormal spindle morphologies and chromosome alignment in mouse oocytes. Oocytes were immnunostained with α-tubulin-FITC antibody to visualize spindle and counterstained with PI to visualize chromosome. Scale bar, 20μm. **B.** The rate of aberrant spindles was recorded in the control, Zfp207-KD and rescue oocytes. **C.** The rate of misaligned chromosomes was recorded in the control, Zfp207-KD and rescue oocytes. Data were presented as mean percentage (mean ± SEM) of at least three independent experiments. Asterisk denotes statistical difference at a *p < 0.05* level of significance.

The kinetochore localization of Zfp207 prompted us to examine its possible function in spindle organization and chromosome alignment. We then employed a morpholino-based gene-silencing approach to deplete Zfp207. Fully-grown GV oocytes were microinjected with control and *Zfp207*-specific morpholinos and arrested in medium supplemented with milrinone for 20 h, allowing enough time to deplete the endogenous Zfp207. Following arrest, the oocytes were washed in milrinone-free medium and cultured to metaphase I stage to analyze the spindle morphology and chromosome alignment. Oocytes were immunostained with anti-tubulin-FITC antibody to visualize the spindles and counterstained with PI for the chromosomes. The staining results showed that a large majority of oocytes exhibited a typical barrel-shape spindle and a well-aligned chromosome on the equatorial plate in the control MO-injected group (Figure 2A). In striking contrast, various types of aberrant spindle morphologies including elongated, shortened, multipolar and collapsed spindles as well as misaligned chromosomes were observed in Zfp207 MO-injected oocytes (Figure 2A). More than 45% of oocytes displayed the disorganized spindles and about 40% of oocytes exhibited misaligned chromosomes compared to less than 10% of defects in controls which might be caused by physical damage of microinjection and milrinone toxicity (Figure 2B, 2C). To rule out the possibility that defective spindle assembly and chromosome alignment was due to the off-target effects of morpholinos, we expressed the Zfp207-mCherry in Zfp207-depleted oocytes by injecting *Zfp207-mCherry* cRNA, and then observed the morphology of spindles and chromosomes. As expected, in the rescue oocytes, the rates of defective spindles and chromosomes were decreased to the levels that were comparable to controls (Figure 2B, 2C). Thus, the results suggest that Zfp207 is important for spindle assembly and chromosome alignment during mouse oocyte meiotic maturation.

**Figure 3:**
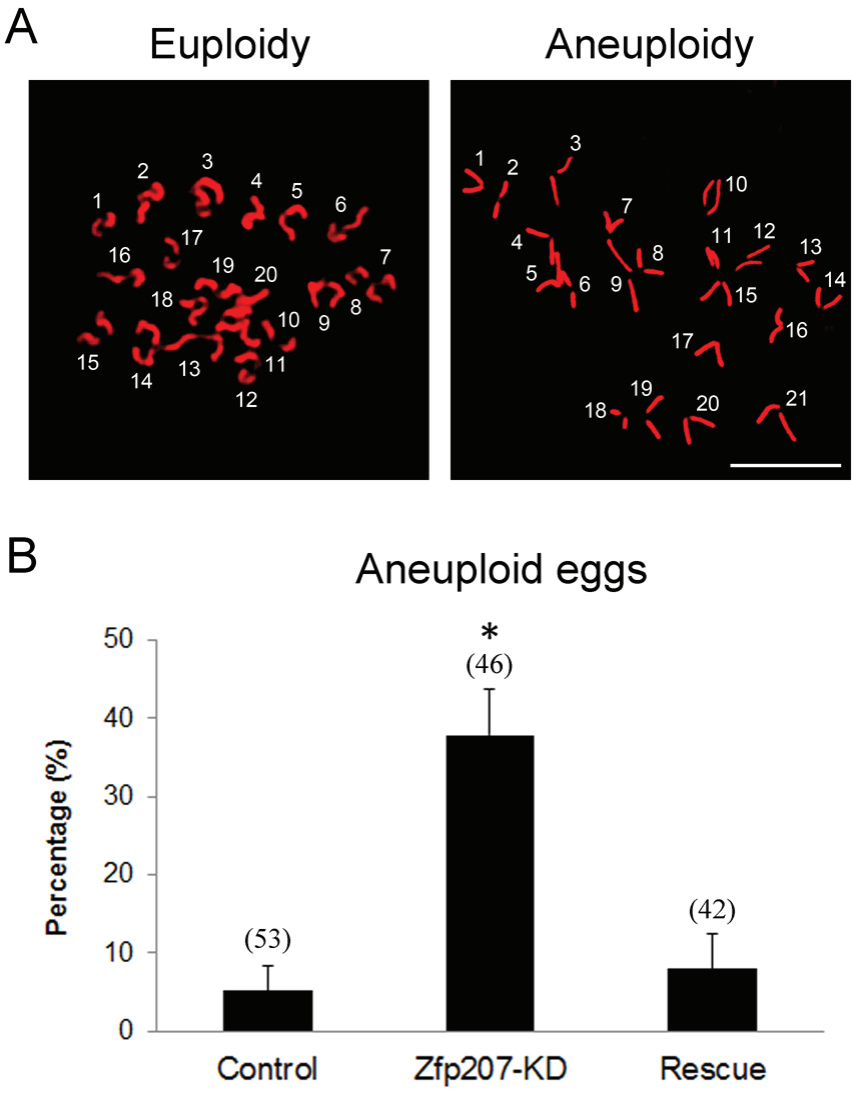
Depletion of Zfp207 generates aneuploid eggs **A.** Representative images of euploid and aneuploid MII eggs. Chromosome spread was performed to calculate the number of chromosomes. Chromosomes were counterstained with PI. Scale bar, 5μm. **B.** The rate of aneuploid eggs was recorded in the control, Zfp207-KD and rescue oocytes. Data were presented as mean percentage (mean ± SEM) of at least three independent experiments. Asterisk denotes statistical difference at a *p < 0.05* level of significance.

Then we asked whether misalignment of chromosomes would produce aneuploidy, an incorrect number of chromosomes in mouse eggs, which might lead to miscarriage, embryonic lethality or genetic disorders. For this purpose, we analyzed the karyotype of metaphase II oocytes by chromosome spreading. As shown in Figure 3A, the number of single chromosomes (univalents) in the normal oocytes was 20, which is the prerequisite for genomic integrity. Whereas a much higher frequency of aneuploid eggs that had more or less 20 univalents occurred in Zfp207-depleted oocytes in comparison with control and rescue oocytes (Figure 3B).

Taken together, these findings suggest that loss of Zfp207 in oocytes are unable to properly assemble the spindles and align the chromosomes and thus prone to produce aneuploid eggs.

### Zfp207 regulates kinetochore-microtubule attachment in oocytes

**Figure 4:**
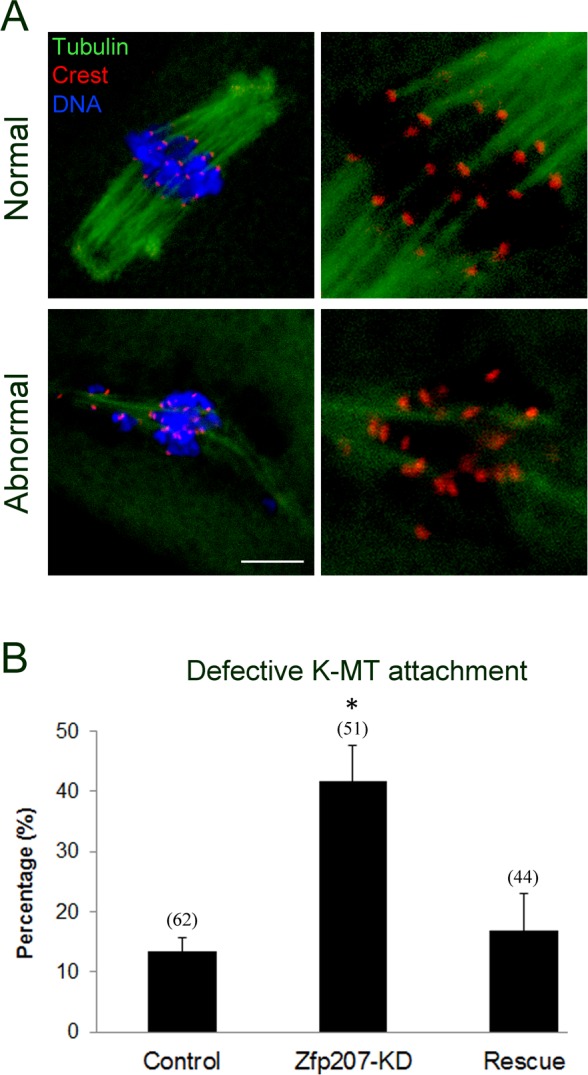
Depletion of Zfp207 disrupts kinetochore-microtubule attachment in mouse oocytes **A.** Representative images of normal and abnormal kinetochore-microtubule attachment in mouse oocytes. Oocytes were immnunostained with α-tubulin-FITC antibody to visualize spindle, with Crest to visualize kinetochore, and counterstained with Hoechst to visualize chromosome. Scale bar, 10μm. **B.** The rate of defective kinetochore-microtubule attachment was recorded in the control, Zfp207-KD and rescue oocytes. Data were presented as mean percentage (mean ± SEM) of at least three independent experiments. Asterisk denotes statistical difference at a *p < 0.05* level of significance.

To determine whether the misalignment of chromosomes upon depletion of Zfp207 was caused by the defective interaction between kinetochores and microtubules, we assessed the stability of kinetochore-microtubule attachment by using cold treatment to depolymerize unstable microtubules that are not attached to kinetochores. To this end, metaphase I oocytes were briefly chilled at 4°C to induce depolymerization of unstable microtubules, and then immunostained with Crest to detect kinetochores, with anti-tubulin-FITC antibody to visualize the spindles and counterstained with Hoechst 33342 for chromosomes. We found that kinetochores became fully attached, chromosomes were well-aligned, and spindles persisted after cold treatment in most of control MO-injected oocytes (Figure 4A, 4B). By contrast, in Zfp207-depleted oocytes, a significantly increased rate of kinetochores with very few cold-stable microtubules was observed compared to controls (Figure 4A, 4B). Whereas in the rescue oocytes expressing Zfp207-mCherry after depletion, the frequency of disrupted kinetochore-microtubule attachment was reduced to the rate comparable to controls (Figure 4B). Collectively, kinetochore-microtubule attachment forms less stably after depletion of Zfp207, which might contribute to the failure of chromosome alignment.

### Depletion of Zfp207 leads to premature polar body extrusion

**Figure 5:**
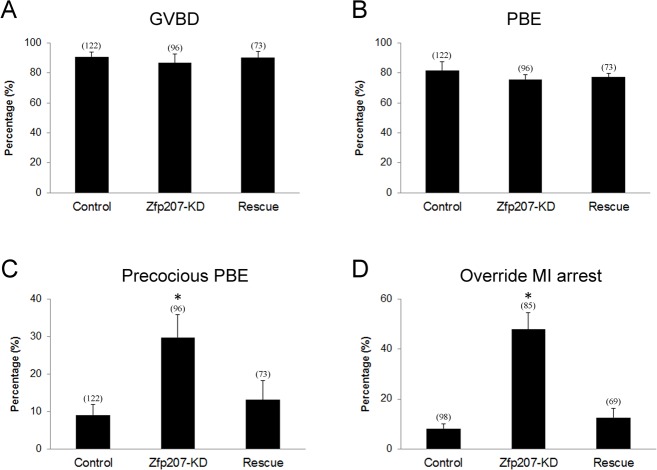
Meiotic progression and SAC activation in Zfp207-depleted oocytes **A.** The rate of germinal vesicle breakdown was recorded in the control, Zfp207-KD and rescue oocytes. **B.** The rate of polar body extrusion was recorded in the control, Zfp207-KD and rescue oocytes. **C.** The rate of precocious polar body extrusion was recorded in the control, Zfp207-KD and rescue oocytes. **D.** The rate of overriding MI arrest was recorded in the control, Zfp207-KD and rescue oocytes. Data were presented as mean percentage (mean ± SEM) of at least three independent experiments. Asterisk denotes statistical difference at a *p < 0.05* level of significance.

Because we have already observed the defective spindle organization, chromosome alignment and kinetochore-microtubule attachment when depleted of Zfp207, we further investigated its possible effects on the oocyte meiotic progression. After culture of GV oocytes to the specific time points, the rates of GVBD and polar body extrusion were calculated in the control, Zfp207-depledted and Zfp207-rescue oocytes, respectively. We found that loss of Zfp207 did not affect either germinal vesicle breakdown or extrusion of first polar body (Figure 5A, 5B), two critical developmental events during oocyte maturation. However, at the time point of 7 h post-GVBD, a higher incidence of PBE was observed in Zfp207-depleted oocytes compared to control and rescue oocytes (Figure 5C), suggesting that PBE occurred earlier in the absence of Zfp207.

The precocious PBE implied that SAC activity was compromised in Zfp207-depleted oocytes. To further confirm this possibility, we tested whether oocytes depleted of Zfp207 would abrogate the metaphase I arrest induced by nocodazole treatment, indicative of SAC inactivation. To this end, GV oocytes were cultured in medium supplemented with 0.04 μg/ml of nocodazole for 12 h to observe the polar body extrusion. The result showed that only about 8% of control and 12% of rescue oocytes could override MI arrest and extrude the first polar body after 12 h of culture. While Zfp207-depleted oocytes displayed a remarkably increased overriding incidence and around 48% of oocytes escaped the MI arrest to reach the MII stage. Taken together, the above results imply that Zfp207 is required for SAC activation and might regulate SAC proteins during meiosis.

### Zfp207 forms a complex with Bub3 and affects its protein level in oocytes

**Figure 6:**
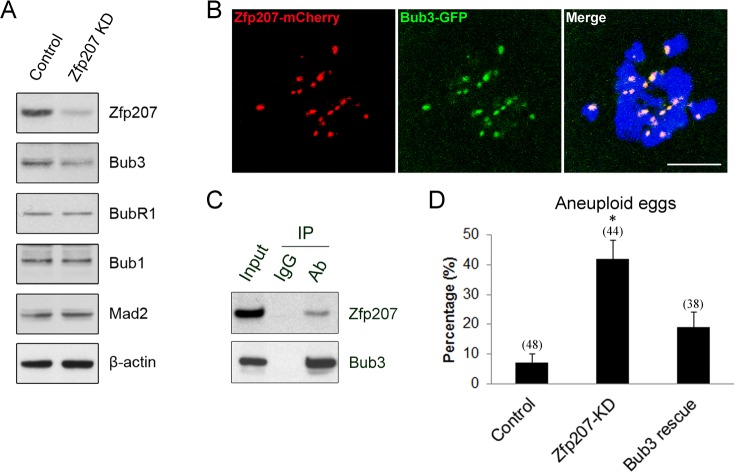
Interaction between Zfp207 and Bub3 in mouse oocytes **A.** Protein levels of SAC components in Zfp207-knockdown oocytes were determined by western blotting. The blots of control and Zfp207-MO injected oocytes were probed with anti-Zfp207, anti-Bub3, anti-BubR1, anti-Bub1, anti-Mad2, and anti-β-actin antibodies, respectively. **B.** Colocalization of Zfp207-mCherry and Bub3-GFP. GV oocytes were co-injected with cRNAs of Zfp207-mCherry and Bub3-GFP, and then cultured to Pro-MI stage. Signals of mCherry and GFP were acquired under confocal microscope at 594nm and 488nm laser, respectively. Chromosomes were counterstained with Hoechst. Scale bar, 5μm. **C.** Zfp207 associates with Bub3 in oocytes. Co-IP was performed to determine the interaction between Zfp207 and Bub3. Oocyte lysates were incubated with IgG and anti-Bub3 antibody, respectively, followed by incubation with protein G beads. The blots of IP eluates were probed with anti-Zfp207 and anti-Bub3 antibodies, respectively. **D.** Quantitative analysis of aneuploid eggs in the control, Zfp207-KD and Bub3-rescue oocytes. Data were presented as mean percentage (mean ± SEM) of at least three independent experiments. Asterisk denotes statistical difference at a *p < 0.05* level of significance.

Since Zfp207 is probably involved in the regulation of SAC function during mouse oocyte meiotic maturation, we next examined the protein levels of several important SAC proteins in Zfp207 MO-injected oocytes by western blotting. The result showed that depletion of Zfp207 did not affect the protein levels of Mad2, Bub1 and BubR1, three vital components of SAC in meiosis, but indeed obviously reduced the protein level of Bub3 (Figure 6A), suggesting that Bub3 might be the downstream molecule that mediates the role Zfp207 in chromosome alignment.

Given that Zfp207 regulates Bub3 in oocytes, we then asked whether Zfp207 interacts with Bub3 as a complex. To address this question, we first performed the cRNA microinjection to detect the localization relationship between Zfp207-mCherry and Bub3-GFP. The overlapping signals of mCherry and GFP revealed that Zfp207 colocalizes with Bub3 at kinetochores (Figure 6B). Moreover, we carried out Co-IP using oocyte lysates with Bub3 antibody. The blot of IP eluate probed by Bub3 antibody showed that Bub3 was specifically present in the antibody group instead of IgG control group (Figure 6C), suggesting that the complex containing Bub3 was pulled down in the eluate. Meanwhile, the blot probed by Zfp207 antibody also showed that Zfp207 appeared only in antibody group (Figure 6C), indicating that Zfp207 form a complex with Bub3 in oocytes.

The higher frequency of aneuploid eggs and decreased protein level of Bub3 in Zfp207-depleted oocytes prompted us to examine their correlation. To this end, we co-expressed Bub3-GFP in Zfp207-MO injected oocytes and then observed the occurrence of aneuploidy. As shown in Figure 6D, overexpression of Bub3 could to a large extent rescue the phenotype of aneuploidy when depleted of Zfp207, confirming the fact that Bub3 works downstream of Zfp207.

## DISCUSSION

The highly conserved spindle assembly checkpoint mechanisms and components have been widely studied in mitosis, whereas the functional roles and components of SAC in meiosis are still not fully clear. Accumulated recent studies have shown that the SAC mechanism indeed operates in meiosis, and that many of its components are conserved between mitosis and meiosis [23, 24]. However, we currently do not know much about the conserved differences between mitosis and meiosis, and even between the male and female meiosis in regulation of SAC [25].

Zinc finger proteins are among the most abundant proteins in eukaryotic genomes and participate in diverse biological eventsas interaction modules that bind DNA, RNA, proteins, or other small molecules [26, 27]. Recent studies in cancer cells have shown that BuGZ is a novel SAC component that is required for chromosome alignment *via* stabilizing Bub3 [11, 14, 16]. However, this mechanism does not work in normal somatic cells. The molecular basis of this selectivity is still an open question. Here, we report that Zfp207 plays a pivotal role in homologous chromosome segregation in mouse oocytes. Although we still cannot address the question why Zfp207 selectively exerts its functions in different cell lines, but our data provide first evidence showing that the function of Zfp207 in regulation of chromosome dynamics is conserved between mitosis and meiosis.

Consistent with the findings in cancer cells, our analyses show that Zfp207 localizes at kinetochores in oocytes after resumption of meiosis, and that depletion of Zfp207 by morpholino injection results in a higher incidence of aberrant spindle morphologies and misaligned chromosomes. These phenotypes are specific to the loss of Zfp207, because it can be rescued by the overexpression of Zfp207-mCherry. A large majority of incorrect chromosome alignment is usually caused by the impaired kinetochore-microtubule attachment, which is also exhibited in Zfp207-depleted oocytes. Our findings show that a significantly increased proportion of kinetochores are unattached by microtubules upon cool treatment which could depolymerize unattached microtubules. Since high-fidelity chromosome segregation prevents aneuploidy and maintains genome stability, our data also reveal that loss of Zfp207 produces a higher frequency of aneuploid eggs which are highly correlated with miscarriage, birth defects and genetic disorders.

Kinetochore-bound SAC proteins such as Mad1, Mad2, Bub1, BubR1, Bub3, and Mps1 generate the wait signal to give cells the time to align all chromosomes to the metaphase plate for equal chromosome segregation [9, 28, 29] [30]. Upon proper alignment of all chromosomes, the SAC signal has to be silenced for metaphase to anaphase transition. Our findings show that depletion of Zfp207 leads to the precocious poly body extrusion and escape of MI arrest induced by nocodazole treatment, two important features indicative of inactivation of SAC activity which have already been characterized in previous studies on SAC components such as Bub1, Bub3, BubR1 and Mad2 in mouse oocytes [3, 31–33]. The protein levels of these SAC components in the absence of Zfp207 have also been detected to figure out how Zfp207 affects SAC activity. In agreement with the observations in cancer cells, we find that protein level of Bub3 is markedly reduced upon knockdown of Zfp207, rather than that of Bub1, BubR1 and Mad2. This suggests that either Zfp207 regulates the protein expression of Bub3 or maintains the stability of Bub3 to ensure proper chromosome alignment in oocytes. Additionally, the colocalization and Co-IP analyses in our study show that Zfp207 binds to Bub3 to form a complex, which further confirms the interaction between Zfp207 and Bub3. Finally, overexpression of Bub3 is able to rescue the aneuploid phenotype of Zfp207-depleted oocytes, suggesting that the role of Zfp207 in regulation of chromosome alignment is, at least, partially mediated by Bub3.

Although several lines of evidence have been provided to demonstrate that Zfp207 is involved in chromosome alignment and activation of SAC during meiosis, many open questions still remain. For example, how does Zfp207 regulate Bub3's protein level in oocytes, through gene expression regulation or protect it from degradation? Is there any other substrate working downstream of Zfp207? Is Zfp207 involved in chromosome segregation in early embryo development? A subsequent research needs to be done to clarify them.

## MATERIALS AND METHODS

### Antibodies

Rabbit polyclonal anti-Zfp207 antibody, sheep polyclonal anti-BubR1 antibody and mouse monoclonal anti-actin antibody were purchased from Abcam (Cambridge, MA, USA; Cat#: ab84802, ab28193 and ab3280); rabbit polyclonal anti-Bub3 antibody was purchased from Santa Cruz Biotechnology (Dallas, TA, USA; Cat#: sc-28258); mouse monoclonal anti-α-tubulin-fluorescein isothiocyanate (FITC) antibody and rabbit polyclonal anti-Bub1 antibody were purchased from Sigma (St. Louis, MO, USA; Cat#: F2168 and B3437); rabbit polyclonal anti-Mad2 antibody was purchased from Covance (Princeton, NJ, USA; Cat#: PRB-452C); Human anti-centromere CREST antibody was purchased from Antibodies Incorporated (Davis, CA, USA; Cat#: 15-234).

### Oocyte collection and culture

Animal care and use were conducted in accordance with the Animal Research Committee guidelines of Nanjing Agricultural University, China.

Female ICR mice (4-6 weeks) were sacrificed by cervical dislocation after intraperitoneal injections of 5 IU pregnant mare serum gonadotropin (PMSG) for 46 hours. Immature oocytes arrested at prophase of meiosis I were collected from ovaries in M2 medium (Sigma, St. Louis, MO, USA). Only those immature oocytes displaying a germinal vesicle (GV) were cultured further in M16 medium under liquid paraffin oil at 37°C in an atmosphere of 5% CO2 in air. At different time points after culture, oocytes were collected for subsequent analysis.

### Morpholino knockdown and cRNA constructs

Fully grown GV-intact oocytes were microinjected with 5-10 pl of non-targeting or Zfp207-targeting morpholinos (Gene tools, Philomath, OR, USA) in M2 medium containing 2.5 μM milrinone. The working concentration of morpholinos was 1 mM. To facilitate the inhibition of mRNA translation by morpholinos, microinjected oocytes were arrested at GV stage in M16 medium containing 2.5 μM milrinone for 20 h, and then transferred to milrinone-free M16 medium to resume the meiosis for further experiments. Zfp207 morpholino sequence: 5′;-CTTCTTCTTGCGACCCATA ACTGCG-3′;.

Full-length of Zfp207 and Bub3 cDNAs were inserted into the pcDNA-3-EGFP vector (Addgene, Cambridge, MA, USA). Capped cRNAs were synthesized from linearized plasmid using T7 mMessage mMachine kit (ThermoFisher, Waltham, MA, USA), and purified with MEGAclear kit (ThermoFisher, Waltham, MA, USA). Typically, 10-12 pl (4% of the oocyte volume) of 0.5-1.0 ug/ul cRNA was injected into oocytes.

### Immunofluorescent and confocal microscopy

Oocytes were fixed in 4% paraformaldehyde in PBS (pH 7.4) for 30 minutes and permeabilized in 0.5% Triton-X-100 for 20 min at room temperature. Then, oocytes were blocked with 1% BSA-supplemented PBS for 1 h and incubated with 1:50-1:100 dilution of primary antibodies at 4°C overnight. After washing four times (5 min each) in PBS containing 1% Tween 20 and 0.01% Triton-X 100, oocytes were incubated with an appropriate secondary antibody for 1 h at room temperature. After washing three times, oocytes were counterstained with PI or Hoechst 33342 (10 μg/ml) for 10 min. Finally, oocytes were mounted on glass slides and observed under a confocal laser scanning microscope (Carl Zeiss 700).

### Immunoprecipitation and immunoblotting analysis

Immunoprecipitation was carried out with rabbit polyclonal anti-Bub3 antibody according to the Instructions for ProFound Mammalian Co-Immunoprecipitation Kit (Pierce, Rockford, IL, USA).

For immunoblotting, oocytes were lysed in 4× LDS sample buffer (ThermoFisher, Waltham, MA, USA) containing protease inhibitor and heated at 95°C for 5 min. Proteins were separated on 12% Bis-Tris precast gels, transferred to PVDF membranes, blocked in 5% nonfat milk in TBS (Tris buffered saline, pH 7.4) with 0.1% Tween 20 (TBST) for 1 h at room temperature, and then probed with 1:500 or 1:1000 dilution of primary antibodies at 4°C overnight. After washing three times in TBST (10 min each), blots were incubated 1 h with a 1:10,000 dilution of HRP (Horse Radish Peroxidase) conjugated secondary antibodies. Chemiluminescence was detected with ECL Plus (Pierce, Rockford, IL, USA) and signals were acquired by Tanon-3900.

### Chromosome spread

Oocytes were exposed to Tyrode's buffer (pH 2.5) for about 30 s at 37°C to remove zona pellucidae. After recovery in M2 medium for 10 min, oocytes were fixed in a drop of 1% paraformaldehyde with 0.15% Triton X-100 on a glass slide. After air drying, chromosomes were counterstained with PI and examined under a laser scanning confocal microscope.

### Statistical analysis

The data were expressed as mean ± SEM and analyzed by one-way ANOVA, followed by LSD's post hoc test, which was provided by SPSS16.0 statistical software. The level of significance was accepted as *p < 0.05*.
